# Screening and evaluation of purines-degrading lactic acid bacteria isolated from traditional fermented foods in Yunnan Province and their uric acid-lowering effects *in vivo*

**DOI:** 10.3389/fmicb.2025.1627956

**Published:** 2025-07-18

**Authors:** Zhen Liu, Xing-Yuan Zou, Jiang Yue, Shan Li, Xia Ou, Chuang Huang, Chen-Jian Liu, Xiao-Ran Li

**Affiliations:** ^1^Faculty of Life Science and Technology, Kunming University of Science and Technology, Kunming, Yunnan, China; ^2^Department of Endocrinology and Metabolism, Renji Hospital, School of Medicine, Shanghai Jiao Tong University, Shanghai, China; ^3^Department of Gastroenterology and Haematology, Anning First People’s Hospital Affiliated to Kunming University of Science and Technology, Kunming, Yunnan, China

**Keywords:** purine, degrading, lactic acid bacteria, uric acid, hyperuricemia

## Abstract

**Introduction:**

Traditional fermented foods have recently been recognized for their potential benefits in managing hyperuricemia (HUA) and gout.

**Methods:**

This study evaluated the purine degradation ability of seventy-eight lactic acid bacteria (LAB) isolated from traditional fermented foods in Yunnan Province, China, by HPLC. The possible mechanisms of *in vitro* purine degradation were explored through whole-genome sequencing, comparative genomics, and qRT-PCR, and the effect of the LAB on HUA in SD rats was verified.

**Results and Discussion:**

*In vitro* results demonstrated that *Limosilactobacillus fermentum* MX-7 and GL-1-3L exhibited high degradation ratios for guanine, while *Pediococcus acidilactici* GJ09-3-7L showed good potential in degrading xanthine. All three strains were also effective in degrading inosine and guanosine. And the genomes of all three strains contained a high number of enzymes related to purine metabolism, transporter and permease. *In vivo* results suggested that the MX-7 strain not only lowering serum uric acid (UA) and urea nitrogen levels in HUA SD rats but also providing a protective effect on renal function. These findings indicate that the MX-7 strain could serve as a promising adjunctive therapy for treating HUA.

## 1 Introduction

Purine is a vital compound in the human body, serving as a key component of nucleic acids, coenzymes, signaling molecules, and energy transfer molecules (Nassogne et al., 2024). It is gradually broken down into uric acid (UA) through the action of xanthine oxidase (Bortolotti et al., 2021; Yang et al., 2022). Hyperuricemia (HUA), the second most common metabolic disorder after diabetes, is closely associated with conditions like gout, chronic kidney disease, and diabetes, posing a significant threat to human health (Lin et al., 2022; Yuan et al., 2022; Zheng et al., 2023). The global incidence of HUA has increased by approximately 20% (Chen-Xu et al., 2019; Cao et al., 2022). According to statistics, the prevalence of HUA among American adults was about 21.4, with 21.2 of men and 21.6% of women affected from 2007–2008 (Zhu et al., 2011). In East China, by 2019, the prevalence ratio for people aged 20–79 was approximately 18.7, with 24.8% of men and 5.6% of women affected, with young men (aged 20–29) emerging as the primary group affected by HUA (She et al., 2022). In the human body, UA is derived from both endogenous and exogenous sources (Li et al., 2018; Liu et al., 2018; Büsing et al., 2019). Endogenous UA, which accounts for 80–90% of the total UA in the body, results from the metabolism of nucleic acids (Li et al., 2018; Liu et al., 2018; Büsing et al., 2019). Exogenous UA, making up 10–20% of the body’s total UA, comes from dietary purine-rich foods, such as organ meats, seafood, meat broths, and sugary beverages (Li et al., 2018; Liu et al., 2018; Büsing et al., 2019). Therefore, modifying dietary habits to reduce the intake of purine-rich foods can help lower UA production, contributing to the prevention and management of HUA (Zhang et al., 2022).

Drug therapy remains the primary approach for lowering serum UA levels. Xanthine oxidase inhibitors, such as allopurinol and febuxostat, work by blocking the conversion of xanthine and hypoxanthine to UA, a process catalyzed by xanthine oxidase (Becker et al., 2005; Schumacher et al., 2008; Bardin and Richette, 2020; Liu G. et al., 2020; Mackenzie et al., 2020; Sekine et al., 2023). However, these drugs may be associated with adverse effects, including skin reactions, diarrhea, nausea, headaches, liver and kidney damage, and eosinophilia (Chen et al., 2019; Dong et al., 2023; Hali et al., 2023; Mohammad et al., 2023; Mao et al., 2025). Other drugs, such as benzbromarone (Benn et al., 2018; Strilchuk et al., 2019) and probenecid (Martínez-Reyes et al., 2020), help promote the excretion of UA and reduce its reabsorption. Nevertheless, benzbromarone has been linked to potential liver toxicity (Kaufmann et al., 2005), while probenecid may cause side effects like skin reactions, headaches, nausea, vomiting, loss of appetite, and gastrointestinal discomfort (Boger and Strickland, 1955; Colín-González Laura and Santamaría, 2013; Garcia-Rodriguez et al., 2023; Mazza et al., 2024). These challenges highlight the urgent need for new therapeutic approaches or alternative drugs (Zhang M. et al., 2023; Mao et al., 2025).

Lactic acid bacteria (LAB) are commonly found in naturally fermented foods (Soemarie et al., 2021). LAB has many benefits, for example, preventing food spoilage (Shehata et al., 2019; Riolo et al., 2024), removing mycotoxins from beverages (Nahle et al., 2022), and enhancing intestinal function and boosting immunity (Soemarie et al., 2021). Recent studies have shown that LAB can participate in the metabolism and degradation of purines, suggesting their potential for developing low-purine foods. For example, Li et al. (2014) demonstrated that *Lactobacillus brevis* DM9218-A can lower serum UA levels in male HUA Wistar rats (30 days old), indicating its potential to prevent and aid in the treatment of HUA. Similarly, Yamada et al. (2017) found that *Lactobacillus gasseri* PA-3 can bind and absorb inosine and related compounds *in vitro*, and *in vivo* studies showed that this strain reduces intestinal absorption of inosine 5’-monophosphate, inosine, and hypoxanthine in 8 weeks old Wistar rats. In another study, Lee et al. (2022) isolated and screened *Lacticaseibacillus paracasei* MJM60396, a strain capable of absorbing 100% of inosine and guanosine from fermented foods. After administering this strain to male HUA C57BL/6 mice (7 weeks old) for 3 weeks, their serum UA levels returned to normal. Further experiments revealed that *L. paracasei* MJM60396 prevents HUA by absorbing purines, reducing UA synthesis, and increasing UA excretion (Lee et al., 2022). Lu et al. (2022) isolated *Limosilactobacillus fermentum* 9–4 from Chinese fermented rice-flour noodles and demonstrated its efficient degradation of inosine and guanosine, suggesting its potential for low-purine food development. Wu et al. (2021) isolated *Limosilactobacillus fermentum* JL-3 from fermented food and showed its potential as an adjunctive treatment for HUA. However, the mechanisms by which LAB degrade purines remain unclear, and further research is needed, including whole-genome sequencing and studies on the expression of relevant enzymes. Yunnan Province is renowned for its diverse and vibrant traditional fermented foods (Liu et al., 2015, 2019; Liu X. F. et al., 2020; Zhang S. et al., 2023; Du et al., 2024), which offer a rich source of functional lactic acid bacteria. However, there has been limited research on the purines-degrading capabilities of lactic acid bacteria isolated from these foods.

The aim of this study was to isolate LAB capable of degrading purines from traditional fermented foods in Yunnan Province, and to explore the potential mechanisms of purines degradation *in vitro* through whole-genome sequencing and qRT-PCR. Additionally, the study sought to evaluate the effects of these LAB strains on HUA in SD rats. This research lays the foundation for further exploration of the microbial resources in Yunnan Province, offers potential candidate strains for developing low-purine foods, and presents a novel approach for the adjunctive treatment of HUA.

## 2 Materials and methods

### 2.1 Preparation of lactic acid bacteria

All LAB strains used in this experiment were from the microbial resource development and application research group, Kunming University of Science and Technology (Kunming, Yunnan, China). They were isolated from traditional fermented foods in Yunnan Province. 78 strains were randomly selected for the determination of purines degradation ability. 16S rRNA gene amplicon sequencing to identify bacteria were performed as described by Loong et al. (2016) (Supplementary Table 1), and primers 27F and 1492R were used for PCR amplification (Eden et al., 1991).

### 2.2 Determination of purines degradation ability of strains *in vitro*

*In vitro* assays, degradation ratios, quantitative standard curves and HPLC conditions were performed as described by Wu et al. (2021). In short, firstly, the washed LAB were incubated with 1 mL guanine, hypoxanthine, xanthine, inosine and guanosine (Yuanye, China) solution (20 μg/mL) respectively, at a concentration of 1 × 10^9^CFU/mL, and incubated at 37°C and 180 rpm for 24 h (inosine and guanosine for 12 h). Secondly, purines were sampled at 0, 6, 12, 18 and 24 h, and nucleosides at 0 min, 20 min, 40 min and 60 min. Thirdly, High temperature and pressure (90°C, 10 min) to obtain the non-viable strains. Finally, sonication to obtain the cell-free extracts of strains (Li et al., 2014), and sonication as described by Feliu et al. (1998). The purines remaining contents were calculated by HPLC (Agilent LC1260, United States).

The conditions for HPLC were as follows: Agilent TC-C18(2) (4.6 mm × 250 mm × 5 μm; United States), the mobile phase for purines were ammonium acetate (LC-MS grade; Thermo Fisher, United States)/methanol (LC-MS grade; Thermo Fisher, United States), 90:10, v/v (nucleosides were 30:10, v/v), while column temperature was 30°C, with wavelength of 254 nm, injection volume of 20 μL and the retention time of 15 min. Three biological replicates were performed for all experiments.

### 2.3 Whole-genome sequencing, assembly, annotation and comparative genomics analysis

*Limosilactobacillus fermentum* MX-7 (NODE: OEZ00020971), GL-1-3L (NODE: OEZ00020969) and *Pediococcus acidilactici* GJ09-3-7L (NODE: OEZ00020970) were sent to Shanghai Yuanxu Biotechnology Co., Ltd. (Shanghai, China), and the second and third generations of whole-genome sequencing were performed using the Illumina NovaSeq PE150 and Oxford Nanopore ONT sequencing platforms, respectively (Chen et al., 2023). Assembly and annotation were performed as described by Chen et al. (2023), Niu et al. (2024), and Liu et al. (2024). Comparative genomic analysis of genes encoding nucleoside permease (Yamauchi et al., 2020), xanthine/uracil permeases, cytosine/uracil/thiamine/allantoin permeases, cytosine deaminase and related metal-dependent hydrolases, xanthine/uracil permeases, purine-nucleoside phosphorylase and guanine deaminase (GDA).

### 2.4 Validation by quantitive reverse transcription-PCR

The *L. fermentum* MX-7, GL-1-3L and *P. acidilactici* GJ09-3-7L were incubated into 50 mL MRS liquid medium at a concentration of 1 × 10^9^CFU/mL, containing 40 μg/mL guanine. After incubated at 37°C and 180 rpm for 24 h, total RNA was extracted using EASYspin Plus Bacterial RNA Extraction Kit (Aidlab, China), and qRT-PCR experiments were performed using HiScript^®^ II One Step qRT-PCR SYBR Green Kit (Vazyme, China). Primer sequences were synthesized by Sangon Biotechnology Co., Ltd. (Shanghai, China). GDA gene (F: CACACCCTCTTAGCCAACGGA, R: GATTATCCATCGCCACACGC). With the 16S rRNA gene as the internal reference (Li et al., 2020), strains cultured without purines were used as the control group, and the 2^–ΔΔCt^ method was utilized to normalize the expression results (Cao et al., 2022). Three biological replicates were performed for all experiments.

### 2.5 Potential probiotic characterization *in vitro*

Acid and bile tolerance, antimicrobial activity and the sensibility to the antibiotics of *Limosilactobacillus fermentum* MX-7 were performed as described by Li et al. (2014). In short, inoculating MX-7 strain in MRS liquid medium (HuanKai Microbial, China) with pH = 2.0 and 3.0. The bile salt (Yuanye, China) solutions at final concentrations of 0.3%. The pathogenic bacteria were *Staphylococcus aureus*, *Escherichia coli*, *Salmonella enterica*, *Salmonella pullorum* and *Listeria monocytogenes*. The contents of antibiotic discs (Binhe, China) were shown in Table 4. Three biological replicates were performed for all experiments.

### 2.6 Animals and experimental design

Thirty specific pathogen-free (SPF) 8–10 weeks old male SD rats were purchased from the Department of Experimental Animal Science, Kunming Medical University (Kunming, Yunnan, China), with one rat per cage. The rats were maintained at 23°C ± 2°C and 50% ± 10% relative humidity, with a 12 h/12 h light/dark cycle. All rats were fed a cobalt-60 irradiated basic feed purchased from Suzhou Shuangshi Experimental Animal Stall Food Technology Co., Ltd. (Suzhou, Jiangsu, China). The experiment was conducted in accordance with the animal management regulations of the Ministry of Science and Technology of China and approved by the Experimental Animal Ethics Committee of the Kunming University of Science and Technology (Approval No. PZWH -KUST-202501040001-1).

**FIGURE 1:**
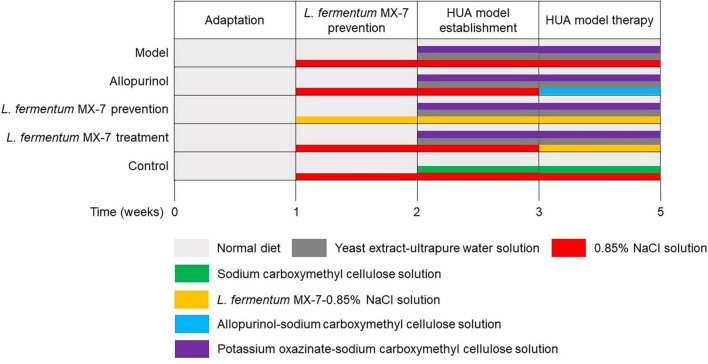
The procedure of the animal experiment. HUA, Hyperuricemia.

Establishment of HUA SD rats model as described by Cao et al. (2022) and Ni et al. (2021). In short, after a 1-week adaptation period, the rats were weighed and divided randomly into five groups (*n* = 6 per group). Figure 1 presents the details of the treatment protocol. In the second week, *L. fermentum* MX-7 prevention group was gavaged with 1 × 10^10^ CFU MX-7 strain-0.85% NaCl solution daily, and the other groups were gavaged with 0.85% NaCl solution. In the third week, the control group was added with sodium carboxymethyl cellulose solution (5 g/L; Yuanye, China), and the other groups were added with yeast extract (15 g/kg; Sangon, China)-ultrapure water solution and potassium oxazinate (250 mg/100 g; Yuanye, China)-sodium carboxymethyl cellulose solution. After successful modeling, allopurinol and MX-7 strain treatment groups replaced 0.85% NaCl solution with allopurinol (4.2 mg/100 g; Yuanye, China)-sodium carboxymethyl cellulose solution and 1 × 10^10^ CFU MX-7 strain-0.85% NaCl solution, respectively. On the 21st, 28th, and 35th days, 1 mL of tail vein blood was collected 60 min after gavage for serum isolation (Ni et al., 2021). After obtaining serum on the 35th day, two rats in each group were randomly selected to be anesthetized with 3% isoflurane and euthanized for cervical dislocation. One kidney per rats was fixed by 4% paraformaldehyde (Servicebio, China) for 24 h (Wu et al., 2021; Lee et al., 2022).

### 2.7 Serum biochemical analysis and histopathological assessment

The collected serum was measured for serum UA, serum urea nitrogen and serum creatinine by BECKMAN COULTER DxC 700 AU (BECKMAN COULTER, United States) automatic biochemical detector (Lee et al., 2022). The renal tissues were embedded in paraffin, sectioned and stained with Hematoxylin and Eosin (H&E), and observed under OLYMPUS BX51 microscope (OLYMPUS, Japan) (Wu et al., 2021; Lee et al., 2022). Six biological replicates were performed for serum biochemical analysis, and two for histopathological assessment.

### 2.8 Statistical analysis

The procedure of the animal experiment and representative arrangement of genomic regions from multiple organisms’ charts were generated using Microsoft PowerPoint 2016. Line and bar charts were generated using GraphPad Prism (v8). The KEGG purine metabolism maps were generated using the KEGG mapper visualization tool. Statistical significance was performed using one-way ANOVA with Tukey’s multiple comparison test with IBM SPSS Statistics (v27). *p* < 0.05 was considered statistically significant. The data was expressed as the mean ± SD.

## 3 Results

### 3.1 Isolation of purines degrading strains

The quantitative standard curves and equations were shown in Supplementary Figure 1 and Supplementary Table 2. We measured the *in vitro* degradation ability of guanine, hypoxanthine and xanthine of seventy-eight strains of LAB (Supplementary Table 3). The best strains for guanine degradation were *L. fermentum* MX-7 and GL-1-3L, with degradation ratios of (82.68 ± 1.76)% and (75.11 ± 1.03)% respectively (Table 1). *Limosilactobacillus fermentum* MX-15 was the best strain to degrade hypoxanthine, but the degradation ratio was only (11.00 ± 0.28)%, almost no degradation ability. *P. acidilactici* GJ09-3-7L had the best degradation ability of xanthine, and the degradation ratio was (31.53 ± 0.43)% (Table 1). We selected three strains (MX-7, GL-1-3L and GJ09-3-7L) with the best degradation effect of guanine and xanthine to degrade inosine and guanosine. The three strains can degrade inosine and guanosine 100% (Table 1).

**TABLE 1:** Degradation ratios of purines (%).

Strains name	Guanine degradation ratio	Xanthine degradation ratio	Inosine degradation ratio	Guanosine degradation ratio
*L. fermentum* MX-7	82.68 ± 1.76	–0.09 ± 0.27	100	100
*L. fermentum* GL-1-3L	75.11 ± 1.03	6.93 ± 1.25	100	100
*P. acidilactici* GJ09-3-7L	5.09 ± 0.95	31.53 ± 0.43	100	100

The negative value in the table indicates that the purines levels in the culture solution increases compared with the control group, which may be due to the production of purines by the strains.

### 3.2 Purines degrading abilities undergo changes *in vitro*

#### 3.2.1 Purines degradation abilities at different time points

**FIGURE 2:**
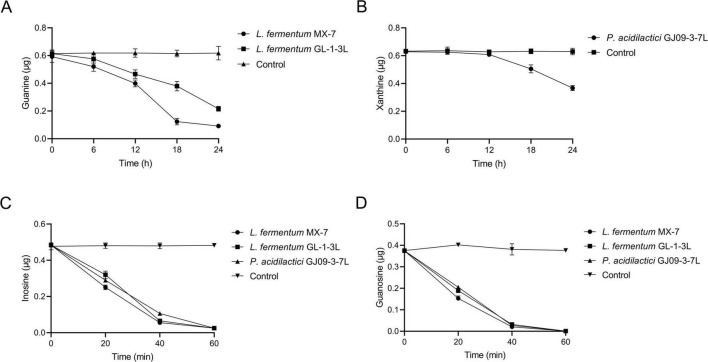
Purines degradation abilities of strains at different time points. **(A)** Guanine, **(B)** Xanthine, **(C)** Inosine, **(D)** Guanosine. Control: Control without strains.

The ability of *L. fermentum* MX-7 to degrade guanine was greater than that of *L. fermentum* GL-1-3L. The degradation ratios of MX-7 and GL-1-3L strains were (84.57 ± 0.01)% and (64.74 ± 0.01)%, respectively, at 24 h (Figure 2A). *P. acidilactici* GJ09-3-7L had no obvious degradation effect on xanthine within 0–12 h, and began to degrade xanthine after 12 h, but the degradation was slow, and the degradation ratio was only (41.59 ± 0.01)% at 24 h (Figure 2B). In addition, the three strains rapidly degraded inosine and guanosine from 0 to 40 min, and tended to be stable from 40 to 60 min. At 60 min, the degradation ratios of inosine and guanosine by the three strains were close to 100% (Figures 2C,D).

#### 3.2.2 Purines degradation abilities between non-viable and viable strains

**FIGURE 3:**
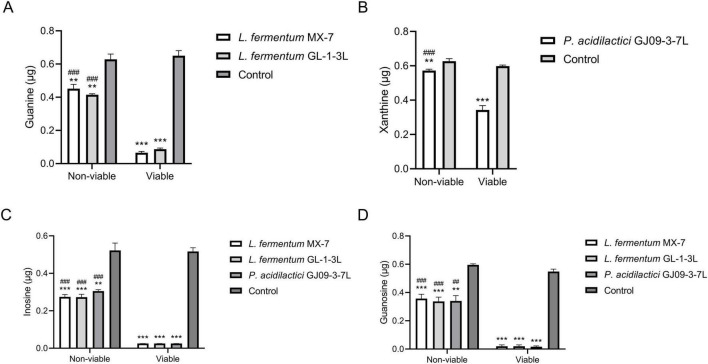
Purines degradation abilities between non-viable and viable strains. Non-viable and viable strains were incubated with guanine and xanthine for 24 h, inosine and guanosine for 60 min, respectively, and the remaining contents were calculated by HPLC. **(A)** Guanine, **(B)** Xanthine, **(C)** Inosine, **(D)** Guanosine. Control: Control without strains. ** *p* < 0.01, *** *p* < 0.001 compared with their respective control group. ## *p* < 0.01, ### *p* < 0.001 compared with their respective viable strains.

The guanine degradation ability of non-viable *L. fermentum* MX-7 and GL-1-3L was significantly lower than that of viable cells (*p* < 0.001) (Figure 3A). The xanthine degradation ability of non-viable *P. acidilactici* GJ09-3-7L was significantly lower than that of viable cells (*p* < 0.001) (Figure 3B). In addition, compared with the viable strains, the degradation ability of the three non-viable strains to inosine (*p* < 0.001) and guanosine (*p* < 0.001, *p* < 0.01) was significantly reduced (Figures 3C,D).

#### 3.2.3 Purines degradation abilities of cell-free extracts

**FIGURE 4:**
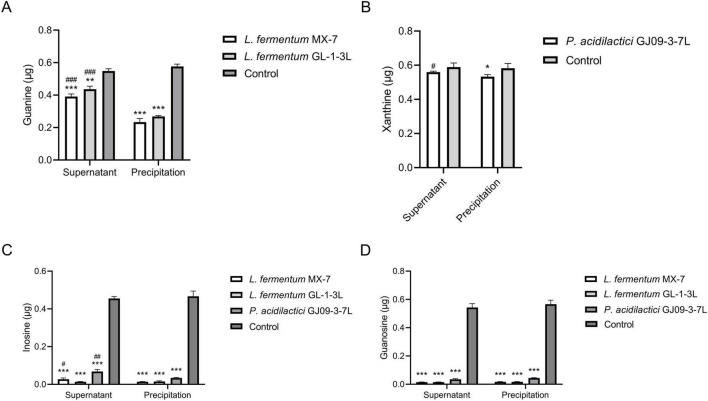
Purines degradation abilities of cell-free extracts. The washed 1 × 10^9^ CFU strains were sonicated and centrifuged to obtain the supernatant and precipitation, respectively. They were incubated with guanine and xanthine for 24 h, inosine and guanosine for 60 min, respectively, and then and the remaining contents were calculated by HPLC. **(A)** Guanine, **(B)** Xanthine, **(C)** Inosine, **(D)** Guanosine. Control: Control without strains. *p < 0.05, **p < 0.01, ***p < 0.001 compared with their respective control group. #*p* < 0.05, ##*p* < 0.01, ###*p* < 0.001 compared with their respective precipitation.

The precipitation of *L. fermentum* MX-7 and GL-1-3L had significantly higher degradation ability of guanine than their supernatant (*p* < 0.001) (Figure 4A). The precipitation of *P. acidilactici* GJ09-3-7L had significantly higher degradation ability of xanthine than its supernatant (*p* < 0.05) (Figure 4B). The degradation of inosine by the precipitation of MX-7 (*p* < 0.05) and GJ09-3-7L (*p* < 0.01) strains were significantly higher than that of their supernatant (Figures 4C,D).

### 3.3 Whole-genome and comparative genomics analysis

**FIGURE 5:**
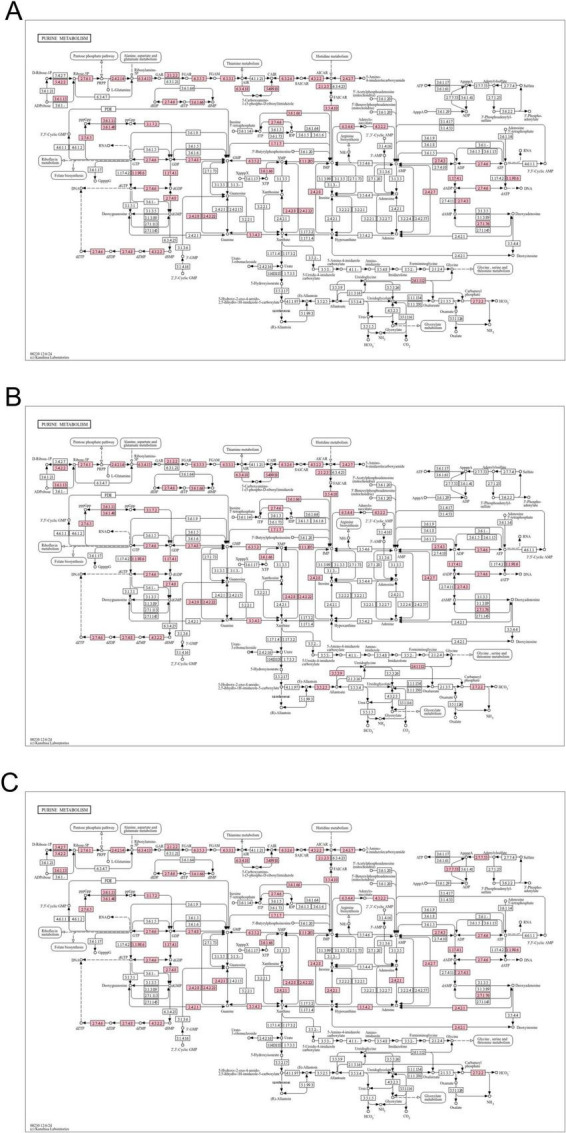
Purine metabolism maps. **(A)** *L. fermentum* MX-7, **(B)** *L. fermentum* GL-1-3L, **(C)** *P. acidilactici* GJ09-3-7L. Pink indicates the presence of the enzyme.

In order to further explore the molecular mechanism of purines degradation by LAB *in vitro*, we sequenced the whole-genome of *L. fermentum* MX-7, GL-1-3L and *P. acidilactici* GJ09-3-7L, which had good degradation effect on purines, in order to further clarify the related enzyme system of purines degradation. The genomic characteristics of the three strains was listed in Supplementary Table 4. As shown in the chromosome circle maps (Supplementary Figure 2), MX-7 strain contained 1 chromosome, GL-1-3L strain contained 1 chromosome and 2 plasmids and GJ09-3-7L strain contained 1 chromosome and 4 plasmids. Supplementary Figure 3 illustrated that, except for unknown functions, most of the COG functional categories genes were enriched in DNA replication, recombination, and repair, amino acid transport and metabolism, translation, ribosomal structure and biogenesis and carbohydrate transport and metabolism. According to the annotation result of KEGG (Supplementary Figure 4), genes responsible for amino acid metabolism or carbohydrate metabolism for the highest proportion. In addition, the KEGG purine metabolism maps showed that the GDA was present in all three strains and the purine-nucleoside phosphorylase was present only in GJ09-3-7L strain (Figure 5). There were five virulence genes in each of the genomes of the three strains (Supplementary Table 5), and there was also a resistance gene in MX-7 and GL-1-3L strains (Supplementary Table 6). Based on the current research results, the three strains could metabolize different types of carbon sources and had good propagation ability, and there were no obvious potential safety problems in the future application of the three strains.

**FIGURE 6:**
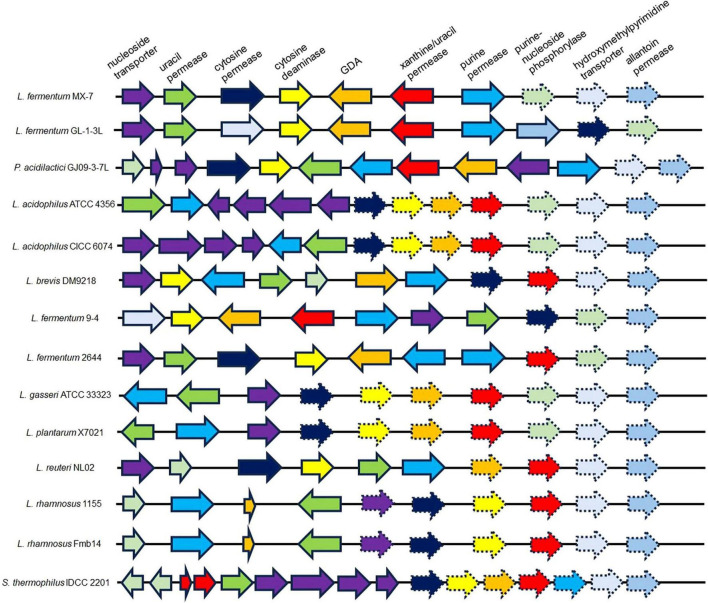
Representative arrangement of genomic regions from multiple organisms that catabolize purines and nucleosides. Solid arrows indicate presence; dashed arrows indicate absence. GDA: guanine deaminase. *Lactobacillus acidophilus* ATCC 4356 (Ma et al., 2023) (CP139575.1, NCBI), *Lactobacillus acidophilus* CICC 6074 (Li et al., 2024) (CP135998.1, NCBI), *Levilactobacillus brevis* DM9218 (Wang H. et al., 2019) (NZ_KZ793387.1, NZ_KZ793388.1, NZ_KZ793389.1, NZ_KZ793390.1, NZ_KZ793391.1, NZ_KZ793392.1, NZ_KZ793393.1, NZ_KZ793394.1, NZ_KZ793395.1 and NZ_KZ793396.1, NCBI), *Limosilactobacillus fermentum* 9-4 (Lu et al., 2022) (NZ_CP076082.1, NCBI), *Limosilactobacillus fermentum* 2644 (Li et al., 2022) (NZ_CP053312.1 and NZ_CP053313.1, NCBI), *Lactobacillus gasseri* ATCC 33323 (Yamada et al., 2017) (NC_008530.1, NCBI), *Lactiplantibacillus plantarum* X7021 (Du et al., 2022; Liu et al., 2022) (NZ_CP025412.1, NZ_CP025413.1, NZ_CP025414.1, NZ_CP025415.1, NZ_CP025416.1, NZ_CP025417.1 and NZ_CP025418.1, NCBI), *Limosilactobacillus reuteri* NL02 (Hu, 2022) (NZ_CP089962.1, NZ_CP089963.1 and NZ_CP089964.1, NCBI), *Lacticaseibacillus rhamnosus* 1155 (Li et al., 2022) (CP048623.1, CP048624.1 and CP048625.1, NCBI), *Lacticaseibacillus rhamnosus* Fmb14 (Zhao et al., 2023) (NZ_CP101845.1 and NZ_CP101846.1, NCBI) and *Streptococcus thermophilus* IDCC 2201 (Al Kassaa et al., 2024) (CP035306.1, NCBI).

Comparative genomics revealed that MX-7, GL-1-3L, and GJ09-3-7L strains have a high number of enzymes related to purine transport and metabolism (Figure 6). The three strains all have cytosine deaminase, GDA and xanthine/uracil permease, which were largely absent in other strains. In addition, the presence of hydroxymethylpyrimidine transporter in the GL-1-3L strain and *Limosilactobacillus fermentum* 9-4, and the presence of allantoin permease only in the GL-1-3L strain demonstrate the unique ability of the GL -1-3L strain.

### 3.4 Quantitive reverse transcription-PCR analysis of guanine deaminase

**FIGURE 7:**
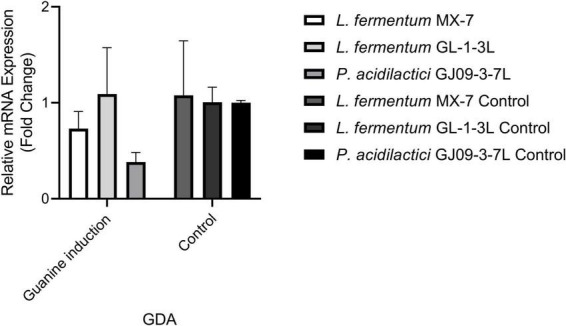
Effects of guanine on the expression level of GDA in strains. GDA: guanine deaminase.

In order to further explore the effect of guanine deaminase on the degradation of guanine, we conducted qRT-RCR analysis. Under the induction of guanine, changes in GDA expression level were observed in *L. fermentum* MX-7, GL-1-3L, and *P. acidilactici* GJ09-3-7L. The GDA levels decreased in MX-7 and GJ09-3-7L strains (Figure 7).

### 3.5 Probiotic properties of *L. fermentum* MX-7

Because *L. fermentum* MX-7 had the highest degradation rate of guanine, in order to evaluate the possibility of MX-7 strain in fermented food, we studied its probiotic properties. The survival ratio of *L. fermentum* MX-7 in MRS liquid medium with pH = 3.0 for 3 h was 89.03%, and the survival ratio in MRS liquid medium with 0.3% bile salt concentration for 3 h was 70.75%, indicating that MX-7 strain has a certain ability of acid and bile salt tolerance (Table 2). Meanwhile, MX-7 strain had different degrees of inhibition on the five pathogenic bacteria, and had strong inhibition on *S. aureus*, *E. coli*, *S. enterica* and *S. pullorum*, but weak inhibition on *L. monocytogenes* (Table 3). In addition, MX-7 strain was resistant to clindamycin, levofloxacin, ciprofloxacin, gentamicin and ceftazidime (Table 4).

**TABLE 2:** Acid and bile salt tolerance of *L. fermentum* MX-7.

*L. fermentum* MX-7	CFU/Ml		Survival ratios (%)
	0 h	3 h	
pH = 2.0	(2.78 ± 0.98) × 10^8^	(3.32 ± 1.32) × 10^4^	–
pH = 3.0	(7.75 ± 1.12) × 10^8^	(6.90 ± 1.03) × 10^8^	89.03
0.3% bile salt	(9.37 ± 1.23) × 10^8^	(6.63 ± 1.37) × 10^8^	70.75
Control	(1.36 ± 1.52) × 10^9^	(1.81 ± 1.21) × 10^9^	133.08

**TABLE 3:** *L. fermentum* MX-7 inhibitory diameter.

Strain name	Inhibitory diameter (mm)				
	*S. aureus*	*E. coli*	*S. enterica*	*S. pullorum*	*L. monocytogenes*
*L. fermentum* MX-7	13.62 ± 0.12	15.56 ± 0.32	20.07 ± 0.55	21.62 ± 0.71	11.69 ± 0.27

**TABLE 4:** The susceptibility of *L. fermentum* MX-7 to different antibiotics.

Antibiotics	Dose (μg/disc)	Inhibitory diameter (mm)	Susceptibility
Clindamycin	2	11.69 ± 0.33	R
Levofloxacin	5	0.00	R
Ciprofloxacin	5	0.00	R
Ampicillin	10	27.33 ± 1.93	S
Gentamicin	10	3.35 ± 0.50	R
Ceftazidime	30	11.62 ± 0.93	R
Cefazolin	30	24.08 ± 0.51	S

S, susceptible, the diameter of inhibition zone ≥ 17 mm; I, intermediate, the diameterof inhibition zone between 12 and 17 mm; R, resistant, the diameter of inhibition zone ≤ 12 mm resistance (Li et al., 2014).

### 3.6 *L. fermentum* MX-7 restored impaired renal function

**FIGURE 8:**
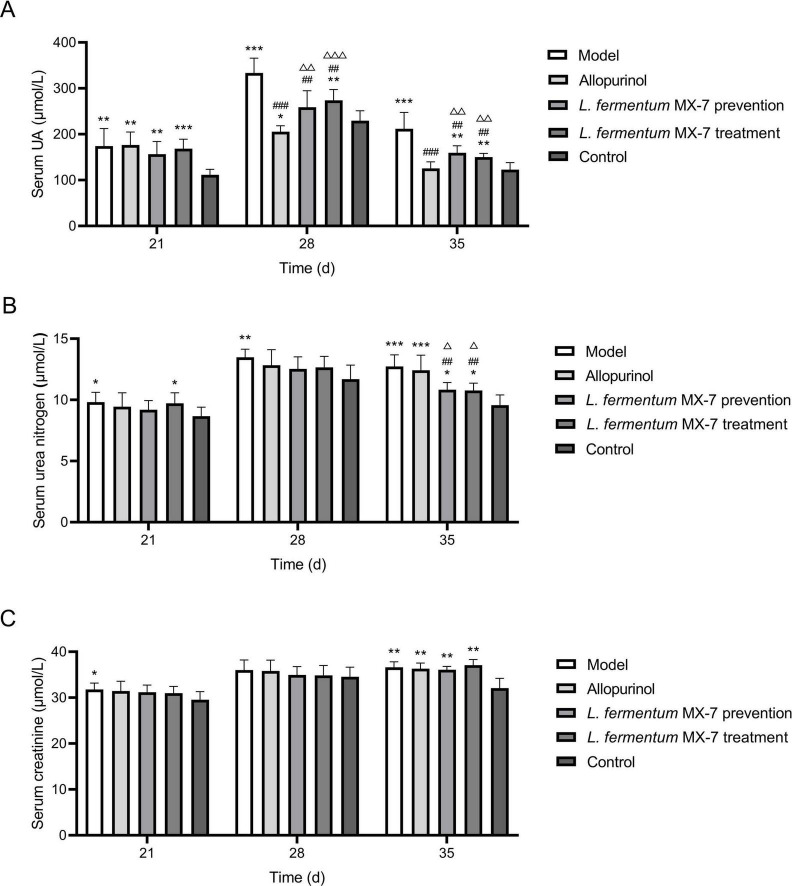
Serum biochemical indexes of rats. **(A)** Serum UA level, **(B)** serum urea nitrogenlevel, **(C)** serum creatinine level. UA, Uric acid. *p < 0.05, **p < 0.01, ***p < 0.001 compared with the control group. ##*p* < 0.01, ###*p* < 0.001 compared with the model group. Δ*p* < 0.05, ΔΔ*p* < 0.01, ΔΔΔ*p* < 0.001 compared with the allopurinol group.

In order to verify the effect of *L. fermentum* MX-7 on HUA SD rats, we performed *in vivo* experiments. Figure 8 showed the changes of serum UA, serum urea nitrogen and serum creatinine in rats. For the serum UA of rats, the serum UA levels of the model (*p* < 0.01), allopurinol (*p* < 0.01), MX-7 strain prevention (*p* < 0.01) and treatment groups (*p* < 0.001) on the 21st day were significantly higher than those of the control group, indicating that the rats HUA model were successfully constructed, and the preventive effect of MX-7 strain on HUA in rats was not obvious. On the 28th and 35th days, we found that the serum UA levels of allopurinol (*p* < 0.001), MX-7 strain prevention (*p* < 0.01) and treatment groups (*p* < 0.01) were significantly lower than that of model group, and the serum UA level of allopurinol group was significantly lower than that of MX-7 strain prevention (*p* < 0.01) and treatment groups (*p* < 0.001, *p* < 0.01), indicating that MX-7 strain can reduce the serum UA level of HUA rats, but it was not as obvious as allopurinol. On the 28th day we also found that elevated serum UA levels in all groups, which could be due to feed and environmental effects. For the serum urea nitrogen of rats, the serum urea nitrogen levels of the model and MX-7 strain treatment groups on the 21st day were significantly higher than those of the control group (*p* < 0.05), indicating the damage effect of HUA on the kidney of rats, and the use of MX-7 strain prevention may reduce the kidney damage. On the 28th day, the level of serum urea nitrogen in the model group was significantly higher than that in the control group (*p* < 0.01). On the 35th day, the serum urea nitrogen levels in the model and allopurinol groups were significantly higher than those in the MX-7 strain prevention (*p* < 0.01, *p* < 0.05), treatment (*p* < 0.01, *p* < 0.05) and control groups (*p* < 0.001), indicating that MX-7 strain has a certain protective effect on the kidney of rats. For the serum creatinine of rats, the serum creatinine level of the model group was significantly higher than that of the control group on the 21st day (*p* < 0.05). On the 35th day, the serum creatinine levels in the model, allopurinol, MX-7 strain prevention and treatment groups were significantly higher than those in the control group (*p* < 0.01).

**FIGURE 9:**
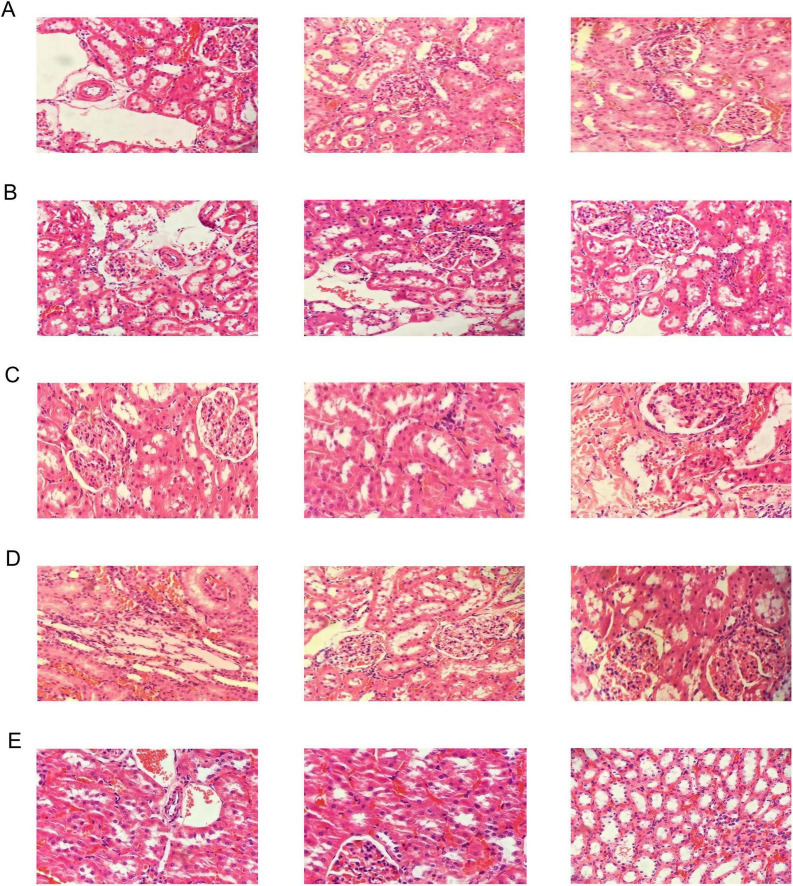
Histopathological analyses of H&E-stained kidney sections [the final magnification was 200X (only for the rightmost image in Figure 9E) or 400X (for the others)]. **(A)** Model group, **(B)** Allopurinol group, **(C)** *L. fermentum* MX-7 prevention group, **(D)** *L. fermentum* MX-7 treatment group, **(E)** Control group.

Compared with the control group, the renal vascular wall in the model and allopurinol groups were significantly thickened, and there was inflammatory cell infiltration in the renal interstitium. The boundary of glomerulus was unclear, and the capillary endothelial cells swelled. The endothelial cells of renal tubules fell off. The MX-7 strain prevention and treatment groups can significantly improve these symptoms, but also observed a small amount of inflammatory cell infiltration in the renal interstitium, swelling of glomerular capillary endothelial cells, and a small amount of renal tubular endothelial cells falling off. Although the reduction of serum UA in the MX-7 strain prevention and the treatment groups were not as obvious as that in the allopurinol group, it can protect the kidney to a certain extent (Figure 9).

## 4 Discussion

In this study, the LAB isolated from traditional fermented foods in Yunnan Province were able to degrade purines *in vitro* (Table 1), and the three strains of LAB screened from them have the potential to develop low purine foods. Among them, *L. fermentum* MX-7 can not only reduce the serum UA and urea nitrogen levels of HUA rats (Figures 8A,B), but also reduce the damage of HUA to the kidney (Figure 9), indicating that MX-7 strain has the potential of adjuvant treatment of HUA. This study filled the gap of the study on the degradation of purines by LAB isolated from traditional fermented foods in Yunnan Province.

We found that the best strains for guanine degradation were *L. fermentum* MX-7 and GL-1-3L, and *P. acidilactici* GJ09-3-7L had the best degradation ability to xanthine (Supplementary Table 1). Our research results were similar to those of Wang et al. (2023), they screened *Limosilactobacillus fermentum* LF-1 from Huangjiu fermentation broth, and the degradation ratios of guanine, hypoxanthine, xanthine, adenine, inosine and guanosine were 95.0, 94.9, 65.9, 95.1, 97.4, and 83.4%, respectively. However, we have not screened LAB with good degradation effect of hypoxanthine, and the highest degradation ratio of xanthine was only (31.53 ± 0.43)% (Supplementary Table 1), which was similar to the research results of Ni et al. (2021). In addition, these three strains can degrade inosine and guanosine 100% (Table 1), which were higher than those of Lu et al. (2022). To further investigate the degradation mechanism, we performed genomic analysis and qRT-PCR. We found that GDA plays a huge role in degrading guanine by comparative genomics analysis and we found GDA in MX-7, GL-1-3L, and GJ09-3-7L strains (Figure 6). *L. acidophilus* CICC 6074 (Li et al., 2024) and *L. plantarum* X7021 (Du et al., 2022; Liu et al., 2022) were both deficient in GDA (Figure 6), and their guanine degradation ratios were less than 40%. In addition, transporter and permease may also play a huge role in purine metabolism in LAB (Martinussen et al., 2010; Liu et al., 2022), whereas many of them were found in MX-7, GL-1-3L, and GJ09-3-7L strains as compared to other (Figure 6). Nucleoside transporter plays a great role in the degradation of nucleosides (Martinussen et al., 2010), and we found it in MX-7, GL-1-3L, and GJ09-3-7L strains (Figure 6). *L. rhamnosus* Fmb14 (Zhao et al., 2023) deficient in nucleoside transporter (Figure 6), and it degrades nucleosides by less than 37% and at a slow ratio. In contrast, strains containing it (Figure 6) (such as *L. acidophilus* CICC 6074 (Li et al., 2024) and *S. thermophilus* IDCC 2201 (Al Kassaa et al., 2024) degraded nucleosides degradation ratios were greater than 50%; MX-7, GL-1-3L, GJ09-3-7L (Table 1), *L. brevis* DM9218 (Wang H. et al., 2019), *L. fermentum* 9-4 (Lu et al., 2022), and *L. reuteri* NL02 (Hu, 2022) greater than 90%) had a higher degradation ratio of nucleosides. Xanthine is a precursor substance for the synthesis of UA. We also found by KEGG purine metabolism map (data not shown) that although none of the 14 strains in the comparative genome (Figure 6) could continue to degrade xanthine, while MX-7 (Figure 8A), *L. acidophilus* ATCC 4356 (Ma et al., 2023), *L. acidophilus* CICC 6074 (Li et al., 2024), *L. brevis* DM9218 (Wang H. et al., 2019), *L fermentum* 2644 (Li et al., 2022), *L. rhamnosus* 1155 (Li et al., 2022), *L. rhamnosus* Fmb14 (Zhao et al., 2023), and *S. thermophilus* IDCC 2201 (Al Kassaa et al., 2024) all reduced serum UA levels in experimental animals, and we believed that the cause may be attributed to two points. The first point was that the strain competes with the intestinal epithelium for uptake of purines *via* transporter and permease, leading to a reduction in their uptake of purines, and thus a reduction in host UA production (Li et al., 2014; Hsu et al., 2019; Wang H. et al., 2019). This suggested an important role for transporter and permease. The second point is that these strains increased the level of UA in the feces, up-regulated the gene expression of UA transporter, ATP-binding cassette subfamily G-2 in colon and jejunum tissues, accelerated the excretion of UA from the intestines, inhibited the activity of xanthine oxidase in liver and serum (Li et al., 2022), restored intestinal flora and increased intestinal short-chain fatty acid content (Al Kassaa et al., 2024). It is the combination of these two reasons that leads to a decrease in serum UA levels. Besides, we also noted that the GDA expression level of MX-7 and GJ09-3-7L strains were decreased under guanine induction, and GL-1-3L strain expression level was similar to the control group (Figure 7), despite they all had the high degradation ratio of guanine except GJ09-3-7L strain (Supplementary Table 1). Although the GJ09-3-7L strain contained GDA (Figure 6), it did not degrade it at a high ratio (Supplementary Table 1), and one of the reasons may be that its GDA expression was inhibited. After the transfer of guanine to MX-7 and GL-1-3L strains *via* transporter and permease, there may be isoenzymes or other degradation pathways or utilization of guanine as an energy source in addition to the action of GDA, but this still needs to be verified by LC-MS. For example, high concentrations of inosine could activate compensatory metabolic pathways of *L. rhamnosus* Fmb14 to catalyze inosine as an energy source and produce intracellular folic acid and riboflavin (Zhao et al., 2023).

LAB can degrade and assimilate purines (Xiao et al., 2020; Kuo et al., 2021; Lu et al., 2022). For degradation, LAB degrade inosine to hypoxanthine and guanosine to guanine (Guimaraes et al., 2011; Kuo et al., 2021; Ni et al., 2021; Lu et al., 2022; Liao et al., 2024). Purines are not as easily absorbed by the intestine as nucleosides (Salati et al., 1984; Stow and Bronk, 1993; Yamada et al., 2016; Kuo et al., 2021), which may increase the excretion of purines in the intestine. LAB can further degrade guanine and hypoxanthine to xanthine (Wang H. et al., 2019; Kuo et al., 2021; Lu et al., 2022; James et al., 2023). For assimilation, LAB absorb purines in the environment and use them for their own growth without producing corresponding degradation products (Xiao et al., 2020; Kuo et al., 2021; Lu et al., 2022). In order to further determine whether purines were degraded or assimilated by the LAB in this study (Li et al., 2014), we analyzed the degradation ability of the cell-free extracts (supernatant and precipitation) of MX-7, GL-1-3L, and GJ09-3-7L strains to purines. The results showed that the cell-free extracts of MX-7 (*p* < 0.001) and GL-1-3L (*p* < 0.01, *p* < 0.001) strains were still able to significantly degrade guanine (Figure 4A), but the degradation ratio was lower than that of viable strains, suggesting that they had both degradation and assimilation effects on guanine. However, only the precipitation of GJ09-3-7L strain was able to significantly degrade xanthine (*p* < 0.05) (Figure 4B), but the degradation ratio was still lower than that of viable strain, indicating that xanthine mainly entered into the intracellular metabolism rather than being directly degraded by enzymes, as evidenced by the fact that the GJ09-3-7L strain degraded xanthine hardly at all from 0 to 12 h, and then degraded it slowly (Figure 2B). We observed that when MX-7, GL-1-3L and GJ09-3-7L strains were thermally inactivated, they could still significantly degrade purines (*p* < 0.01) and nucleosides (*p* < 0.001, *p* < 0.01), but significantly less than the viable strains (*p* < 0.001, *p* < 0.01) (Figure 3). This may indicate that there were still some viable strains under this thermally treatment (90°C, 10 min), which further emphasized the importance of active enzymes and cellular metabolism for purine degradation. At the same time, the cell-free extracts of these three strains were still able to significantly degrade inosine and guanosine (*p* < 0.001) (Figures 4C,D), suggesting that they mainly play a degrading role, i.e., the enzymes in the supernatants and precipitations contacted with inosine and guanosine to directly produce the corresponding degradation products, which is consistent with Li et al. (2014) using *L. brevis* DM9218. However, purine-nucleoside phosphorylase, which is capable of degrading inosine and guanosine, was present in the genome of DM9218 strain (Li et al., 2014; Figure 6), but the enzymes for degrading inosine and guanosine were deficiency in both MX-7 and GL-1-3L strains (Figure 5). From the KEGG purine metabolism map (data not shown), *L. acidophilus* CICC 6074 (Li et al., 2024) and *L. fermentum* 9-4 (Lu et al., 2022) were also both deficient in enzymes for degrading inosine and guanosine, but *in vitro* experiments showed that both strains were able to degrade them, which seems to suggest the presence of isozymes or other pathways that play a role in the degradation, but this needs further confirmation.

When selecting potential probiotic strains, it is important to detect their virulence genes and resistance genes (Papadimitriou et al., 2015; Dlamini et al., 2019). We found virulence genes in MX-7, GL-1-3L, and GJ09-3-7L strains (Supplementary Table 5), which was consistent with the existing studies on LAB (Togay et al., 2010; Dlamini et al., 2019). However, Bennedsen et al. (2011) did not find virulence genes in all LAB they studied. The existence of virulence genes does not guarantee the expression of genes, and there is a great possibility that these genes are not expressed or only weakly expressed (Dlamini et al., 2019). We also found resistance genes in MX-7 and GL-1-3L strains (Supplementary Table 6). Dlamini et al. (2019) found resistance genes in *Limosilactobacillus reuteri* VB4 and *Streptococcus salivarius* NBRC13956. But in many cases, resistance genes are not transferable (Dlamini et al., 2019). Therefore, we believe that MX-7, GL-1-3L and GJ09-3-7L strains have the potential to become probiotics.

Since MX-7 strain has the highest degradation ratio of guanine (Supplementary Table 1), its probiotic properties were evaluated to determine whether it can survive in the gastrointestinal tract and colonize successfully, laying the foundation for the development of low purine foods in the future. The gastrointestinal tract is rich in digestive enzymes and has strong acidity (Li et al., 2014). Bile is produced by the liver and contributes to the digestion of lipids in the small intestine (Li et al., 2014). Bile salts are present in bile (Li et al., 2014). Acid environment and bile salts can inhibit bacterial growth (Li et al., 2014). We found that MX-7 strain was better than most existing studies in acid resistance, but has poor bile salt resistance (Table 2; Li et al., 2014; Lu et al., 2022; Hussain et al., 2024; Liao et al., 2024). This indicates that MX-7 strain can survive in the intestine. In addition, the antibacterial ability of LAB mainly comes from two aspects. The first is to produce lactic acid and other acidic substances to reduce the environmental pH, making it unsuitable for the growth of other bacteria (Li et al., 2014). The second is the production of bacteriocin and other antibacterial substances (Li et al., 2014). Compared with existing studies (Li et al., 2014), we found that MX-7 strain has stronger ability to inhibit *S. enterica* and *S. pullorum* (Table 3). We also found that MX-7 strain was resistant to clindamycin, levofloxacin, ciprofloxacin, gentamicin and ceftazidime (Table 4), which was similar to the results of Li et al. (2014).

Since experimental animals (such as rats) can convert UA into allantoin for excretion, it is difficult to establish animal HUA model (Sánchez-Lozada et al., 2007; Sanchez-Lozada et al., 2008; Xiao et al., 2020). In this study, we established HUA rats model using yeast extract and potassium oxazinate (Figure 1; Yamada et al., 2017). Compared with the model group, the use of MX-7 strain for prevention and treatment can significantly reduce the serum UA level of HUA rats (*p* < 0.01), but it was still higher than that of allopurinol group (Figure 8A), which was consistent with the results of Li et al. (2014). Allopurinol can directly inhibit xanthine oxidase, thereby directly preventing the production of UA (Li et al., 2014). However, MX-7 strain may reduce UA production by competing with the intestinal epithelium for purine uptake and probiotic effects (Li et al., 2014; Hsu et al., 2019; Wang H. et al., 2019). Therefore, some purines can still be absorbed by the intestinal epithelium and eventually metabolized into UA (Li et al., 2014; Hsu et al., 2019; Wang H. et al., 2019). HUA can lead to the destruction of renal structure and function, and increase the levels of blood urea nitrogen and creatinine, which are indicators of renal toxicity (Lee et al., 2022). On the 35th day, we found that the serum urea nitrogen (*p* < 0.05) and serum creatinine (*p* < 0.01) levels in the MX-7 strain prevention and treatment groups were still significantly higher than those in the control group (Figures 8B,C), which is consistent with the research results of Lee et al. (2022). This may indicate that HUA will cause some damage to the kidney of rats in MX-7 strain prevention and treatment groups. Nevertheless, we still found that the prevention and treatment of MX-7 strain can reduce kidney damage to a certain extent through H&E staining of rats kidney (Figure 9), which was consistent with the existing results (Ni et al., 2021; Wu et al., 2021; Cao et al., 2022; Lee et al., 2022). Whether prolonging the intervention time of MX-7 strain can reverse the renal damage caused by HUA is also worth discussing. LAB have a protective effect on renal injury caused by acute HUA (Minelli et al., 2004; Xiao et al., 2020), so we speculate that MX-7 strain may repair the damaged kidney by reducing the production of serum UA and/or urea nitrogen (Ni et al., 2021), restoring intestinal flora and increasing intestinal short-chain fatty acid content (Al Kassaa et al., 2024).

## 5 Conclusion

Here, we screened *L. fermentum* MX-7 and GL-1-3L with high guanine efficiency and *P. acidilactici* GJ09-3-7L with high xanthine degradation efficiency from traditional fermented foods in Yunnan Province by HPLC. And these three strains can efficiently degrade inosine and guanosine. The presence of many purine metabolism, transporter and permease genes revealed the potential of these three strains to degrade purines. The use of MX-7 strain can successfully reduce the serum UA and urea nitrogen levels of HUA SD rats, and can reduce the damage of HUA to the kidney of rats. However, we did not study the extent of assimilation and degradation products of LAB to purines *in vitro*, nor did we set up a control group of non-degrading LAB *in vivo*. Our research provided three candidate strains for the development of low purine foods, and MX-7 strain had the potential of adjuvant therapy for HUA.

## Data Availability

The data that support the findings of this study are available in NODE at https://www.biosino.org/node, accession number OEZ00020969, OEZ00020970, and OEZ00020971.
