# Supplementary material for: Screening and evaluation of purines-degrading lactic acid bacteria isolated from traditional fermented foods in Yunnan Province and their uric acid-lowering effects in vivo
Source: Front Microbiol. 2025 Jul 18;16:1627956. doi: 10.3389/fmicb.2025.1627956 (PMC12313681; doi:10.3389/fmicb.2025.1627956)
Supplement: Supplementary file 1 [file Data_Sheet_1.docx]

**Supplementary Materials (Tables)**

Supplementary Table 1 Sources and species of experimental strains.

| Strain number | Source of strains | 16S rRNA gene amplicon sequencing identification | |
| --- | --- | --- | --- |
|  |  | Species identification | Homology (%) |
| XGL-4 | Milk dregs | *Lactiplantibacillus plantarum* | 100 |
| XGL-5 | Milk dregs | *Lactiplantibacillus plantarum* | 100 |
| XGL-6 | Milk dregs | *Lactiplantibacillus plantarum* | 100 |
| XGL-8 | Milk dregs | *Lactiplantibacillus plantarum* | 100 |
| XGL-10 | Milk dregs | *Lactiplantibacillus plantarum* | 100 |
| XGL-18 | Milk dregs | *Lactiplantibacillus plantarum* | 100 |
| XGL-22 | Milk dregs | *Lactiplantibacillus plantarum* | 100 |
| XGL-24 | Milk dregs | *Lactiplantibacillus plantarum* | 100 |
| XGL-65 | Milk dregs | *Lactiplantibacillus plantarum* | 100 |
| XGL-68 | Milk dregs | *Lactiplantibacillus plantarum* | 100 |
| XGL-72 | Milk dregs | *Lactiplantibacillus plantarum* | 100 |
| XGL-74 | Milk dregs | *Lactiplantibacillus plantarum* | 100 |
| XGL-82 | Milk dregs | *Lactiplantibacillus plantarum* | 100 |
| XGL-91 | Milk dregs | *Lactiplantibacillus plantarum* | 100 |
| XGL-112 | Milk dregs | *Lactiplantibacillus plantarum* | 100 |
| XGL-139 | Milk dregs | *Lactiplantibacillus plantarum* | 100 |
| XGL-144 | Milk dregs | *Lactiplantibacillus plantarum* | 100 |
| XGL-145 | Milk dregs | *Lactiplantibacillus plantarum* | 100 |
| XGL-149 | Milk dregs | *Lactiplantibacillus plantarum* | 100 |
| HH3-2L | Douchi | *Lactiplantibacillus plantarum* | 100 |
| HH09-3-2L | Douchi | *Lactiplantibacillus plantarum* | 100 |
| SP8-6L | Douchi | *Lactiplantibacillus plantarum* | 100 |
| SP8-7L | Douchi | *Lactiplantibacillus plantarum* | 100 |
| QB3-1 | Douchi | *Lactiplantibacillus plantarum* | 100 |
| YM4-3 | Douchi | *Lactiplantibacillus plantarum* | 100 |
| QB3-2 | Douchi | *Lactiplantibacillus plantarum* | 100 |
| QB3-3 | Douchi | *Lactiplantibacillus plantarum* | 100 |
| SP-8 | Douchi | *Lactiplantibacillus plantarum* | 100 |
| SP09-2-8L | Douchi | *Lactiplantibacillus plantarum* | 100 |
| YY09-2-1L | Douchi | *Lactiplantibacillus plantarum* | 99 |
| KY-6-3L | Douchi | *Lactiplantibacillus plantarum* | 100 |
| YY09-3-1L | Douchi | *Lactiplantibacillus plantarum* | 98 |
| HH4-2 | Douchi | *Lactiplantibacillus plantarum* | 100 |
| AY01 | Douchi | *Lactiplantibacillus plantarum* | 100 |
| XGL-97 | Milk dregs | *Limosilactobacillus fermentum* | 100 |
| XGL-120 | Milk dregs | *Limosilactobacillus fermentum* | 100 |
| XGL-128 | Milk dregs | *Limosilactobacillus fermentum* | 100 |
| XGL-131 | Milk dregs | *Limosilactobacillus fermentum* | 100 |
| XGL-136 | Milk dregs | *Limosilactobacillus fermentum* | 100 |
| XGL-155 | Milk dregs | *Limosilactobacillus fermentum* | 100 |
| MX-2 | Sour Rice Noodles | *Limosilactobacillus fermentum* | 100 |
| MX-7 | Sour Rice Noodles | *Limosilactobacillus fermentum* | 100 |
| MX-15 | Sour Rice Noodles | *Limosilactobacillus fermentum* | 100 |
| MX-22 | Sour Rice Noodles | *Limosilactobacillus fermentum* | 100 |
| GL-1-3L | Douchi | *Limosilactobacillus fermentum* | 100 |
| MX-5 | Sour Rice Noodles | *Leuconostoc lactis* | 100 |
| MX-6 | Sour Rice Noodles | *Leuconostoc lactis* | 100 |
| MX-8 | Sour Rice Noodles | *Leuconostoc lactis* | 100 |
| MX-11 | Sour Rice Noodles | *Leuconostoc lactis* | 100 |
| MX-14 | Sour Rice Noodles | *Leuconostoc lactis* | 100 |
| MX-16 | Sour Rice Noodles | *Leuconostoc lactis* | 100 |
| MX-19 | Sour Rice Noodles | *Leuconostoc lactis* | 100 |
| MX-24 | Sour Rice Noodles | *Leuconostoc lactis* | 100 |
| MX-29 | Sour Rice Noodles | *Leuconostoc lactis* | 100 |
| JSL3-1 | Douchi | *Weissella paramesenteroides* | 100 |
| JSL3-2 | Douchi | *Weissella paramesenteroides* | 99 |
| JSL3-3 | Douchi | *Weissella paramesenteroides* | 99 |
| JSL3-4 | Douchi | *Weissella paramesenteroides* | 100 |
| JSL3-5 | Douchi | *Weissella paramesenteroides* | 100 |
| JSL3-6 | Douchi | *Weissella paramesenteroides* | 100 |
| JSL3-7 | Douchi | *Weissella paramesenteroides* | 100 |
| JSL3-8 | Douchi | *Weissella paramesenteroides* | 100 |
| JSL3-9 | Douchi | *Weissella paramesenteroides* | 100 |
| JSL3-10 | Douchi | *Weissella paramesenteroides* | 100 |
| JSL3-12 | Douchi | *Weissella paramesenteroides* | 100 |
| JSL5-2 | Douchi | *Weissella paramesenteroides* | 100 |
| JSL5-3 | Douchi | *Weissella paramesenteroides* | 100 |
| JSL5-1 | Douchi | *Weissella confusa* | 100 |
| JSL7-1 | Douchi | *Weissella confusa* | 100 |
| BZ06 | Douchi | *Lactococcus lactis* | 100 |
| JS5-2 | Douchi | *Lactococcus lactis* | 100 |
| GJ09-2-2L | Douchi | *Pediococcus pentosaceus* | 100 |
| GJ09-4-2L | Douchi | *Pediococcus pentosaceus* | 100 |
| GJ09-4-6L | Douchi | *Pediococcus pentosaceus* | 100 |
| GJ09-5-1L | Douchi | *Pediococcus pentosaceus* | 100 |
| JS4-2 | Douchi | *Pediococcus acidilactici* | 100 |
| GJ09-3-7L | Douchi | *Pediococcus acidilactici* | 100 |
| AS02 | Douchi | *Lacticaseibacillus casei* | 100 |

Supplementary Table 1 (continued)

Supplementary Table 2 Quantitative standard curve equation.

| Name | Retention time (min) | Standard curve equation | R^2^ |
| --- | --- | --- | --- |
| Guanine | 5.053 | y = 435.43x + 0.9246 | 0.9999 |
| Hypoxanthine | 5.071 | y = 4807.9x + 4.2492 | 1.0000 |
| Xanthine | 6.782 | y = 1133.6x + 7.6106 | 0.9991 |
| Inosine | 7.027 | y = 2126x - 52.995 | 0.9990 |
| Guanosine | 7.627 | y = 2500.4x - 2.7798 | 1.0000 |

Supplementary Table 3 Degradation ratios of purines by lactic acid bacteria.

| Strain name | Guanine degradation ratio (%) | Hypoxanthine degradation ratio (%) | Xanthine degradation ratio (%) |  |
| --- | --- | --- | --- | --- |
| *L. plantarum* XGL-4 | -12.52±1.66 | -1.29±0.18 | 0.38±0.32 |  |
| *L. plantarum* XGL-5 | 39.77±0.53 | 4.20±0.18 | -5.96±0.21 |  |
| *L. plantarum* XGL-6 | 21.67±1.30 | 9.45±4.85 | 5.75±0.13 |  |
| *L. plantarum* XGL-8 | 11.62±2.19 | 10.08±2.45 | 3.04±0.26 |  |
| *L. plantarum* XGL-10 | 48.30±0.71 | 4.11±0.15 | 2.28±0.10 |  |
| *L. plantarum* XGL-18 | 17.88±0.37 | -1.33±0.12 | 5.02±0.16 |  |
| *L. plantarum* XGL-22 | 15.41±1.01 | -2.18±0.06 | 1.34±0.35 |  |
| *L. plantarum* XGL-24 | 51.73±0.40 | 5.04±0.16 | 0.82±0.17 |  |
| *L. plantarum* XGL-65 | -10.90±0.72 | -1.02±0.11 | 4.64±0.21 |  |
| *L. plantarum* XGL-68 | -15.27±0.61 | -1.62±0.06 | 3.36±0.37 |  |
| *L. plantarum* XGL-72 | -9.09±0.99 | -4.17±0.07 | -0.17±0.34 |  |
| *L. plantarum* XGL-74 | -8.49±1.48 | 8.00±2.58 | 2.47±0.46 |  |
| *L. plantarum* XGL-82 | 28.66±1.64 | 5.79±2.32 | 3.43±0.30 |  |
| *L. plantarum* XGL-91 | 23.32±1.44 | 7.45±2.60 | 3.60±0.26 |  |
| *L. plantarum* XGL-112 | 36.59±1.23 | 9.88±0.07 | 2.97±0.11 |  |
| *L. plantarum* XGL-139 | 21.50±1.14 | 8.61±0.23 | 3.83±0.29 |  |
| *L. plantarum* XGL-144 | 15.4±1.36 | -2.45±0.28 | 5.81±0.49 |  |
| *L. plantarum* XGL-145 | -13.52±1.18 | -6.08±0.13 | -4.01±0.22 |  |
| *L. plantarum* XGL-149 | -16.87±0.67 | -7.90±0.06 | 1.40±0.34 |  |
| *L. plantarum* HH3-2L | 32.05±0.43 | 4.16±0.19 | -0.17±0.31 |  |
| *L. plantarum* HH09-3-2L | 24.58±0.86 | 3.02±0.20 | 1.43±0.32 |  |
| *L. plantarum* SP8-6L | 23.97±0.63 | 1.35±0.18 | 1.25±0.25 |  |
| *L. plantarum* SP8-7L | 6.60±0.82 | 0.40±0.10 | 3.44±0.39 |  |
| *L. plantarum* QB3-1 | 11.23±0.72 | 2.73±0.21 | 3.93±0.52 |  |
| *L. plantarum* YM4-3 | 13.77±0.55 | 1.96±0.02 | 22.71±0.84 |  |
| *L. plantarum* QB3-2 | 3.01±0.15 | 0.47±0.03 | 4.50±0.05 |  |
| *L. plantarum* QB3-3 | 13.17±1.50 | 5.71±0.19 | 4.05±0.33 |  |
| *L. plantarum* SP-8 | 11.48±0.27 | 3.80±0.05 | 16.61±0.05 |  |
| *L. plantarum* SP09-2-8L | 9.60±0.26 | 1.38±0.14 | 10.34±0.12 |  |
| *L. plantarum* YY09-2-1L | 15.45±0.71 | -2.25±0.20 | 2.48±0.45 |  |
| *L. plantarum* KY-6-3L | 19.85±0.20 | 0.02±0.28 | -2.71±0.11 |  |
| *L. plantarum* YY09-3-1L | | -0.10±0.70 | 7.04±0.16 | 10.16±0.17 |
| *L. plantarum* HH4-2 | | 10.38±1.01 | 0.68±0.20 | 2.43±0.29 |
| *L. plantarum* AY01 | | 30.49±1.25 | 8.27±0.29 | 5.88±0.17 |
| *L. fermentum* XGL-97 | | 56.21±1.32 | 7.85±0.09 | -5.97±0.33 |
| *L. fermentum* XGL-120 | | 54.31±0.31 | 9.91±0.23 | 5.96±0.25 |
| *L. fermentum* XGL-128 | | 17.67±1.33 | 1.45±0.12 | 2.50±0.10 |
| *L. fermentum* XGL-131 | | 14.35±1.06 | 2.98±0.20 | 7.69±0.22 |
| *L. fermentum* XGL-136 | | 51.16±0.65 | 10.30±0.09 | 5.04±0.21 |
| *L. fermentum* XGL-155 | | 54.86±2.80 | 6.17±0.05 | -4.23±0.38 |
| *L. fermentum* MX-2 | | 26.35±1.73 | 0.08±0.19 | 8.80±0.18 |
| *L. fermentum* MX-7 | | 82.68±1.76 | 5.82±0.29 | -0.09±0.27 |
| *L. fermentum* MX-15 | | 52.81±0.44 | 11.00±0.28 | 7.45±0.40 |
| *L. fermentum* MX-22 | | 34.40±1.27 | 8.98±0.16 | 2.35±0.28 |
| *L. fermentum* GL-1-3L | | 75.11±1.03 | 4.27±0.03 | 6.93±1.25 |
| *L. lactis* MX-5 | | 37.69±1.78 | 4.53±0.07 | 9.44±0.65 |
| *L. lactis* MX-6 | | 12.94±0.89 | 9.33±0.21 | 2.95±0.27 |
| *L. lactis* MX-8 | | 31.69±1.49 | 4.13±0.22 | 1.10±0.36 |
| *L. lactis* MX-11 | | 5.54±0.31 | 4.18±0.20 | -4.84±0.22 |
| *L. lactis* MX-14 | | 22.05±1.24 | 5.17±0.23 | 3.99±0.54 |
| *L. lactis* MX-16 | | 14.66±0.98 | 9.95±0.17 | 2.44±0.35 |
| *L. lactis* MX-19 | | 12.06±1.08 | 8.03±0.21 | -0.19±0.33 |
| *L. lactis* MX-24 | | 9.44±2.68 | 8.22±0.17 | 9.03±0.46 |
| *L. lactis* MX-29 | | 20.26±1.26 | 5.61±0.17 | 1.64±0.28 |
| *W. paramesenteroides* JSL3-1 | | 15.57±0.37 | 4.29±0.15 | 0.55±0.31 |
| *W. paramesenteroides* JSL3-2 | | 14.32±0.81 | 4.20±0.15 | 3.81±0.58 |
| *W. paramesenteroides* JSL3-3 | | 8.37±0.52 | 2.71±0.20 | 0.47±0.13 |
| *W. paramesenteroides* JSL3-4 | | -0.74±0.75 | 1.28±0.08 | 1.01±0.35 |
| *W. paramesenteroides* JSL3-5 | | 15.99±0.72 | 4.82±0.11 | 1.48±0.34 |
| *W. paramesenteroides* JSL3-6 | | 3.97±0.22 | 0.37±0.03 | 14.80±0.05 |
| *W. paramesenteroides* JSL3-7 | | 29.59±0.18 | 2.69±0.01 | 14.44±0.30 |
| *W. paramesenteroides* JSL3-8 | | 2.30±0.39 | -1.04±0.08 | 1.21±0.30 |
| *W. paramesenteroides* JSL3-9 | | 3.83±0.09 | 9.53±0.04 | 24.47±0.08 |
| *W. paramesenteroides* JSL3-10 | | 13.71±0.23 | 5.61±0.07 | 3.74±0.16 |
| *W. paramesenteroides* JSL3-12 | | 12.69±0.36 | 6.18±0.09 | 1.74±0.39 |
| *W. paramesenteroides* JSL5-2 | | 47.46±0.47 | 9.84±0.10 | 18.69±0.54 |
| *W. paramesenteroides* JSL5-3 | | 49.21±0.38 | 9.96±0.21 | 0.72±0.07 |
| *W. confusa* JSL5-1 | | -11.76±1.3 | 0.55±0.06 | -0.12±0.54 |
| *W. confusa* JSL7-1 | | 26.81±1.30 | 9.76±0.15 | 0.02±0.21 |
| *L. lactis* BZ06 | | 26.46±0.43 | 7.88±0.19 | 3.77±0.16 |
| *L. lactis* JS5-2 | | 10.83±1.01 | 1.35±0.03 | 2.31±0.32 |
| *P .pentosaceus* GJ09-2-2L | | 5.01±0.05 | 4.52±0.07 | 11.74±0.11 |
| *P .pentosaceus* GJ09-4-2L | | 9.24±0.48 | 8.54±0.19 | 4.94±0.40 |
| *P .pentosaceus* GJ09-4-6L | | 10.08±0.36 | 3.37±0.01 | 10.69±0.06 |
| *P .pentosaceus* GJ09-5-1L | | 27.33±0.56 | 1.42±0.29 | 1.15±0.42 |
| *P. acidilactici* JS4-2 | | 14.74±1.24 | -2.14±0.02 | -0.16±0.36 |
| *P. acidilactici* GJ09-3-7L | | 5.09±0.95 | 3.92±0.14 | 31.53±0.43 |
| *L. casei* AS02 | | 0.33±0.79 | 3.22±0.91 | 2.31±0.73 |

The negative value in the table indicates that the purines levels in the culture solution increases compared with the control group, which may be due to the production of purines by the strains.

Supplementary Table 3 (continued)

Supplementary Table 4 The genomic feature of *L. fermentum* MX-7, GL-1-3L and *P. acidilactici* GJ09-3-7L.

| Features | *L. fermentum* MX-7 (NODE: OEZ00020971) | *L. fermentum* GL-1-3L (NODE: OEZ00020969) | *P. acidilactici* GJ09-3-7L (NODE: OEZ00020970) |
| --- | --- | --- | --- |
| Genomic size (bp) | 2,062,813 | 2,201,332 | 2,094,431 |
| GC content (%) | 51.81 | 51.28 | 41.94 |
| Coding genes | 2,033 | 2,177 | 2,020 |
| tRNA | 58 | 58 | 56 |
| 5S rRNA | 5 | 5 | 5 |
| 16S rRNA | 5 | 5 | 5 |
| 23S rRNA | 5 | 5 | 5 |

Supplementary Table 5 Virulence genes.

| Strain name | NCBI_ID | Function |
| --- | --- | --- |
| *L. fermentum* MX-7 | WP_003682571.1 | Chaperonin GroEL |
| *L. fermentum* MX-7 | WP_003682593.1 | MULTISPECIES: UTP--glucose-1-phosphate uridylyltransferase GalU |
| *L. fermentum* MX-7 | WP_003682627.1 | MULTISPECIES: ATP-dependent Clp protease proteolytic subunit |
| *L. fermentum* MX-7 | WP_014562272.1 | Elongation factor Tu |
| *L. fermentum* MX-7 | WP_012391767.1 | NADP-dependent phosphogluconate dehydrogenase |
| *L. fermentum* GL-1-3L | WP_003682593.1 | MULTISPECIES: UTP--glucose-1-phosphate uridylyltransferase GalU |
| *L. fermentum* GL-1-3L | WP_003682571.1 | chaperonin GroEL |
| *L. fermentum* GL-1-3L | WP_003682627.1 | MULTISPECIES: ATP-dependent Clp protease proteolytic subunit |
| *L. fermentum* GL-1-3L | WP_003682059.1 | MULTISPECIES: elongation factor Tu |
| *L. fermentum* GL-1-3L | WP_023467422.1 | MULTISPECIES: NADP-dependent phosphogluconate dehydrogenase |
| *P. acidilactici* GJ09-3-7L | WP_053905931.1 | Chaperonin GroEL |
| *P. acidilactici* GJ09-3-7L | WP_002829932.1 | UTP--glucose-1-phosphate uridylyltransferase GalU |
| *P. acidilactici* GJ09-3-7L | WP_002829919.1 | ATP-dependent Clp endopeptidase proteolytic subunit ClpP |
| *P. acidilactici* GJ09-3-7L | EFL96108.1 | Phosphogluconate dehydrogenase (decarboxylating) |
| *P. acidilactici* GJ09-3-7L | WP_002830466.1 | Elongation factor Tu |

Supplementary Table 6 Resistance genes.

| Strain name | NCBI_ID | Function |
| --- | --- | --- |
| *L. fermentum* MX-7 | WP_070447233.1 | Undecaprenyl-diphosphate phosphatase |
| *L. fermentum* GL-1-3L | EEI21492.1 | Undecaprenyl-diphosphatase UppP |

**Supplementary Materials (Figures)**

Supplementary Figure 1


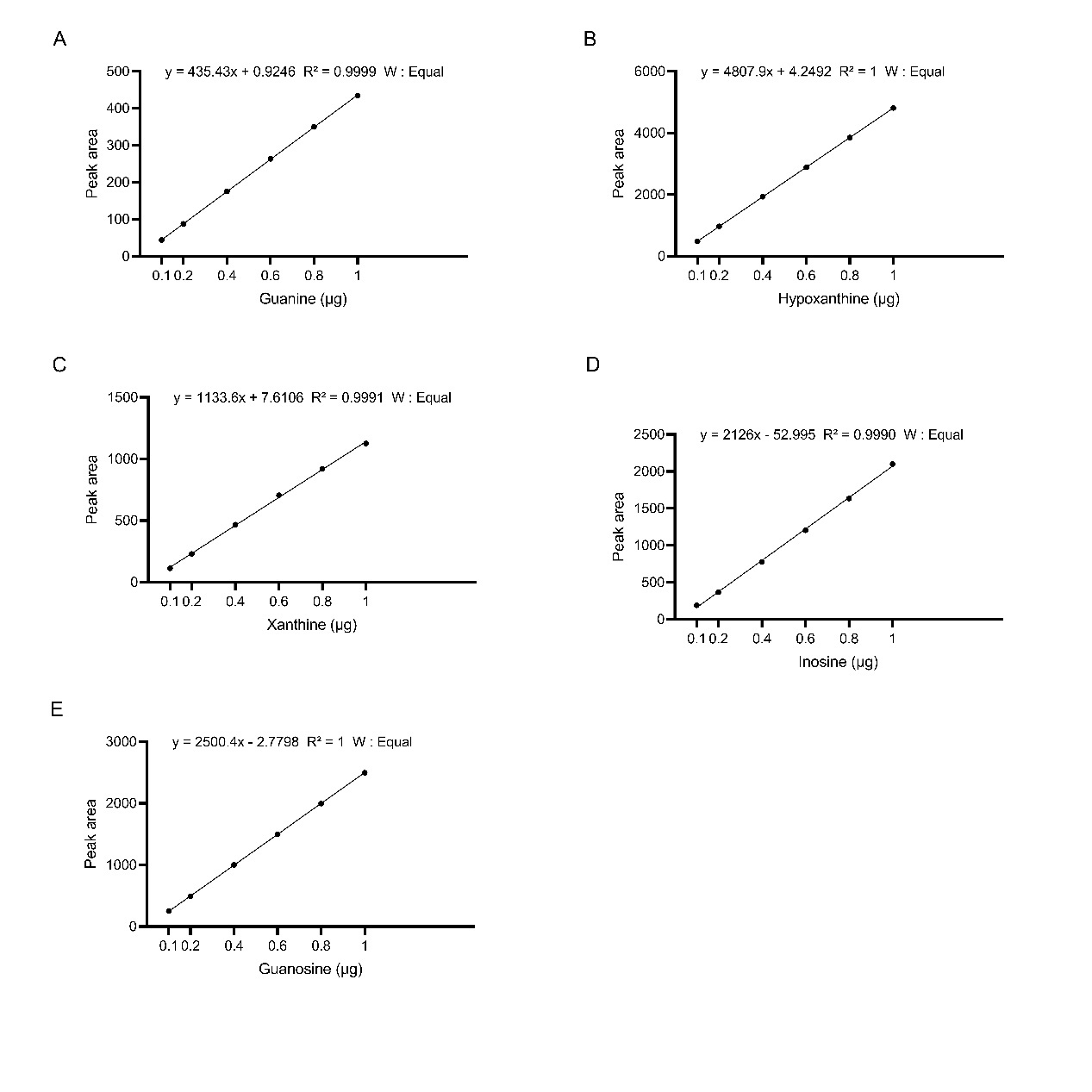


Supplementary Figure 1 Quantitative standard curves. (A) Guanine, (B) Hypoxanthine, (C) Xanthine, (D) Inosine, (E) Guanosine.

Supplementary Figure 2

**
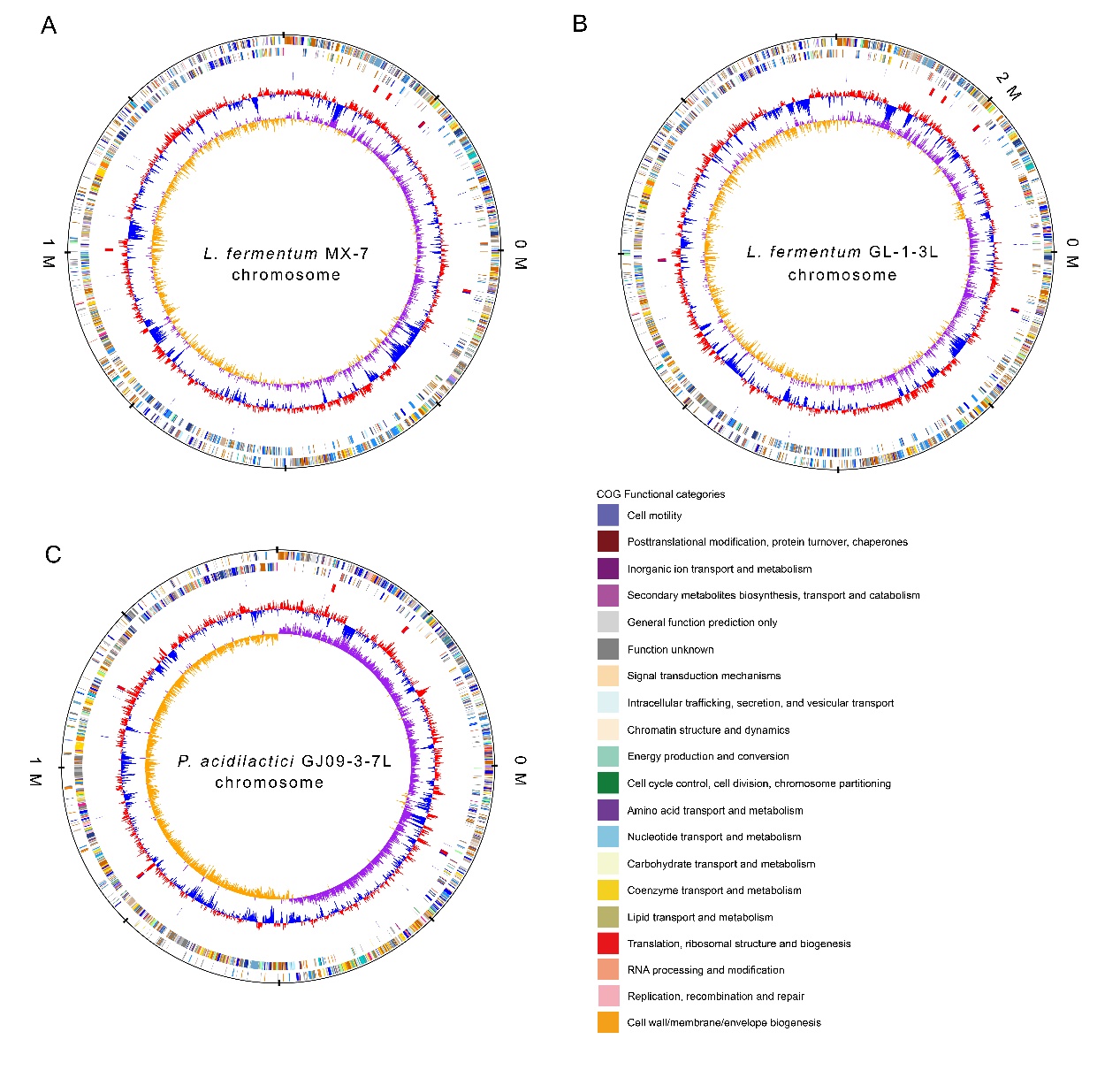
**

Supplementary Figure 2 Chromosome circle maps. (A) *L. fermentum* MX-7, (B) *L. fermentum* GL-1-3L, (C) *P. acidilactici* GJ09-3-7L. The outermost and second circles of the circle diagram are the coding sequence (CDS) on the positive and negative strands, with different colors indicating the functional classification of the different Clusters of Orthologous Groups (COGs) of the CDS; the third circle is for rRNA (red) and tRNA (blue); the fourth circle is for GC content (red>the average GC content of genome, blue<the average GC content of genome); the innermost circle represents the GC skew value ((G-C)/(G+C), purple>0, yellow<0).

Supplementary Figure 3


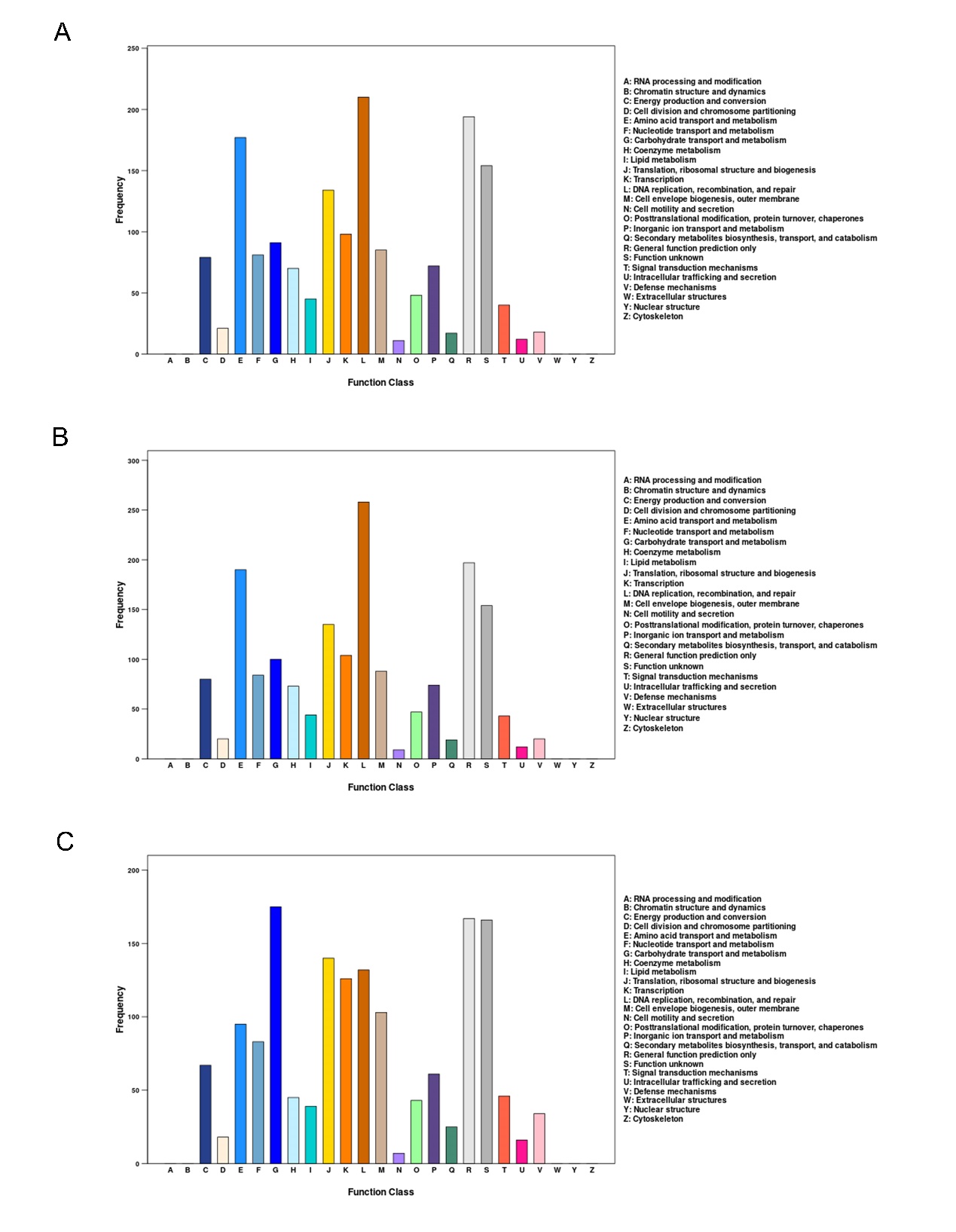


Supplementary Figure 3 The genes of the genome in COG functional categories. (A) *L. fermentum* MX-7, (B) *L. fermentum* GL-1-3L, (C) *P. acidilactici* GJ09-3-7L.

Supplementary Figure 4


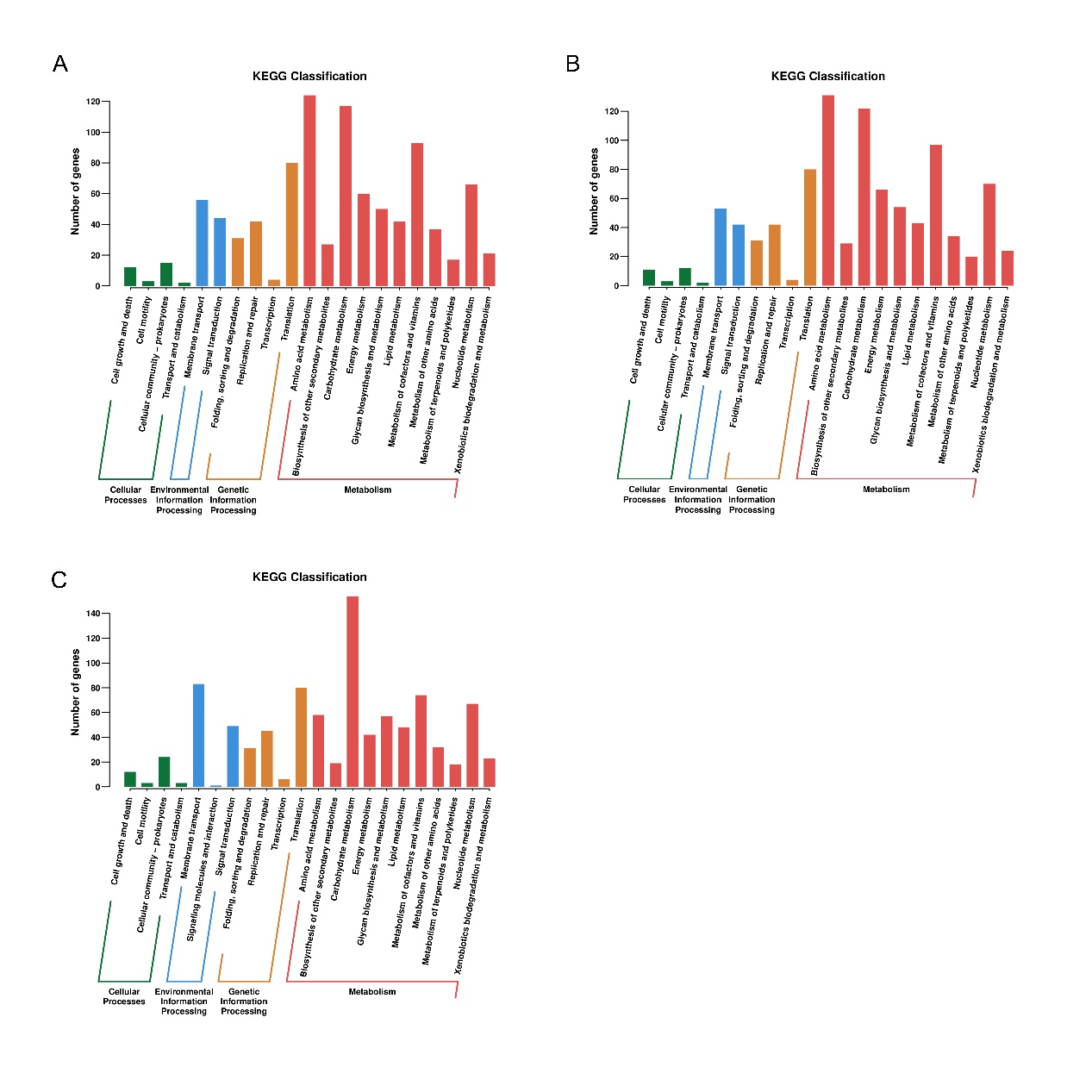


Supplementary Figure 4 The genes of the genome functional annotation in the KEGG metabolic pathway. (A) *L. fermentum* MX-7, (B) *L. fermentum* GL-1-3L, (C) *P. acidilactici* GJ09-3-7L.
